# On the Best Means of Promoting Union by First Intention

**Published:** 1877-05

**Authors:** E. W. Lee

**Affiliations:** Chicago


### ON THE BEST MEANS OF PROMOTING UNION BY FIRST INTENTION.

By E. W. LEE, M. D., Chicago.

Ninety-nine practitioners out of a hundred will proceed to dress an incised or lacerated wound by bringing the edges together, and maintaining them in position—or trying to—by means of strips of adhesive plaster or interrupted sutures of silk. For several years I have been in the habit of using needle sutures for all wounds, varying the size and shape according to the location and depth of the wound. For all wounds not very deep, I use Sharp's No. 12 cambric needle. It is very small, and is easily introduced and extracted.

If plaster be used, no matter how carefully it may be applied, in a few hours it stretches, permitting the edges of the wound to gape although the apposition was perfect when leaving the hands of the surgeon. If interrupted sutures of silk be used in the ordinary way, the edges of the wound are brought together, leaving underneath a cavity for the accumulation of discharges and subsequent suppuration; the silk causes more or less irritation immediately, it begins to cut, and unless taken out in 24 hours, leaves an ugly mark at the seat of the suture. In all wounds over one-third of an inch in length, I use these needle sutures. We all know what an unirritating substance steel is. Needles have entered the body and remained there for years, causing no inconvenience whatever, coming out in an entirely different location from where they had entered. Suppose we have a wound to dress, say one and a half inches long. I proceed in the following manner: Carefully cleanse the part of all foreign matter, *and wait for hemorrhage to cease.* Then if the location and depth of the wound be suitable, take a No. 12 cambric needle in a needle holder, insert it a proper distance from the edge of the wound, push it through at about half the depth of the wound, bring the point out about the same distance on the opposite side.

Take now a piece of stout ligature silk or thread, and surround the transfixed tissue and draw the edges of the wound together. Put in as many sutures as may be necessary to secure perfect apposition, and the dressing is complete. It is useless to put on plasters in addition; they stretch, they are unsightly and unclean. In dressing wounds by this method pressure can be made so as to bring the edges of the wound together *from top to bottom*; no space is left for secretions to accumulate; no chance is left for stretching, and for the edges of the wound to gape; the pressure being so equally distributed, the suture does not cut through as a silk one will. The only objection to allowing the sutures to remain for four or five days, is that after forty-eight hours they are difficult of extraction. This difficulty I have overcome by having the needles electroplated with silver. To extract the needle, I take the end in the needle holder, gently turn it round in the wound once or twice, and then withdraw it. I do not cut the silk, it remains adherent, the blood and serum forming an incrustation, holding the silk in position; this I am careful not to disturb. I once dressed an incised wound 24 inches long, in the manner described. Between 40 and 50 needles (No. 12) were used; every portion of the wound healed by first intention. The advantages of this plan do not by any means end here. Suppose the radial, temporal, or palmar arteries be wounded; many practitioners not expert will spend considerable valuable time in seeking and ligating any of these vessels, and consequently more loss of blood than need be is occasioned. Here the needle suture is not only the best means of bringing the edges of the wound together, but it is the quickest, easiest, and safest means of stopping the hemorrhage by acupressure. I have repeatedly adopted this plan in all the above-mentioned accidents, and always with the utmost satisfaction. Suppose union by first intention does not take place; then cut the silk, withdraw the needles and the amount of retraction that takes place will not be nearly so great as it would had they not been used. I usually succeed in getting union by first intention, and when I have failed it has been either from a faulty condition of the system, or from being too hasty in the application of the dressing. In incised wounds about the neck and face, where primary union is so desirable, this plan is peculiarly suitable. In scalp wounds, prudent practitioners hesitate to use silk sutures, so apt are they to set up erysipelatous inflammation; to make plaster adhere it is absolutely necessary to shave the scalp for a considerable space around the wound. Use needle sutures, and it is not necessary to remove any hair at all, and they may remain in the scalp as long as may be necessary with impunity. This may seem a very small matter to say so much about; but with most of us, dressing wounds is an every-day occurrence, and any improvement that may be introduced, however small, is of practical importance. I have tried this plan so long and thoroughly, and with so much gratification to myself and patients, that I feel it a duty to urge its substitution for silk and plaster entirely. It is not, of course, original with me, yet it is not adopted to any extent by the profession. I am confident that if the dressing be carefully done by those adopting this method, the attending success will be so uniform as to prohibit the employment of any other.

